# Transcriptional response of *P. pastoris *in fed-batch cultivations to *Rhizopus oryzae *lipase production reveals UPR induction

**DOI:** 10.1186/1475-2859-6-21

**Published:** 2007-07-16

**Authors:** David Resina, Mónika Bollók, Narendar K Khatri, Francisco Valero, Peter Neubauer, Pau Ferrer

**Affiliations:** 1 Departament d'Enginyeria Química, Escola Tècnica Superior d'Enginyeria, Universitat Autònoma de Barcelona, 08193-Bellaterra, Spain; 2 Bioprocess Engineering Laboratory, Department of Process and Environmental Engineering and Biocenter Oulu, University of Oulu, Finland; 3 SOLVO Biotechnology, Budaörs, Hungary

## Abstract

**Background:**

The analysis of transcriptional levels of the genes involved in protein synthesis and secretion is a key factor to understand the host organism's responses to recombinant protein production, as well as their interaction with the cultivation conditions. Novel techniques such as the sandwich hybridization allow monitoring quantitatively the dynamic changes of specific RNAs. In this study, the transcriptional levels of some genes related to the unfolded protein response (UPR) and central metabolism of *Pichia pastoris *were analysed during batch and fed-batch cultivations using an X-33-derived strain expressing a *Rhizopus oryzae *lipase under control of the formaldehyde dehydrogenase promoter (*FLD1*), namely the alcohol oxidase gene *AOX1*, the formaldehyde dehydrogenase *FLD1*, the protein disulfide isomerase *PDI*, the *KAR2 *gene coding for the BiP chaperone, the 26S rRNA and the *R. oryzae *lipase gene *ROL*.

**Results:**

The transcriptional levels of the selected set of genes were first analysed in *P. pastoris *cells growing in shake flask cultures containing different carbon and nitrogen sources combinations, glycerol + ammonium, methanol + methylamine and sorbitol + methylamine. The transcriptional levels of the *AOX1 *and *FLD1 *genes were coherent with the known regulatory mechanism of C1 substrates in *P. pastoris*, whereas *ROL *induction lead to the up-regulation of *KAR2 *and *PDI *transcriptional levels, thus suggesting that ROL overexpression triggers the UPR. This was further confirmed in fed-batch cultivations performed at different growth rates. Transcriptional levels of the analysed set of genes were generally higher at higher growth rates. Nevertheless, when ROL was overexpressed in a strain having the UPR constitutively activated, significantly lower relative induction levels of these marker genes were detected.

**Conclusion:**

The bead-based sandwich hybridization assay has shown its potential as a reliable instrument for quantification of specific mRNA species in *P. pastoris *cells grown in fed-batch cultures. As a proof-of-principle, the influence of the carbon and nitrogen sources, the specific growth rate, as well as the ROL overexpression on the transcriptional levels of a reduced set of bioprocess-relevant genes has been quantitatively studied, revealing that ROL overexpression and secretion seems to trigger the UPR in *P. pastoris*, resulting in a physiological bottleneck for the production process.

## Background

The methylotrophic yeast *Pichia pastoris *has emerged as an important production host for both industrial protein production and basic research [1,2]. However, the limited number of systematic metabolic and physiologic characterization studies under bioprocess-relevant conditions currently hampers progress in strain improvement and rational design and optimization of cultivation conditions for heterologous protein production in the *Pichia *system. Information on heterologous gene expression and production of the proteins at different physiological states of the cells is scarce. Furthermore, limited information is available on the cellular responses to heterologous protein production in *P. pastoris*.

Importantly, the *P. pastoris *genome has been recently deciphered, offering innumerable possibilities to pursue coordinated understanding of cellular processes leading to rational cell factory engineering of *P. pastoris*. In this context, new techniques have been recently developed for the quantitative analysis of bioprocess-relevant RNAs. One of those is a magnetic bead-based sandwich hybridisation system, based on two specific oligonucleotide probes [3]. This system is advantageous over the conventional methods, Northern blot or slot blot, due to the high specificity and sensitivity, which are eventually improved by the use of unlabelled helper probes [4]. Furthermore, this system can be automated for high throughput analysis, and is also applicable with minor modifications for the analysis of antigens which is a major advantage in comparison to other techniques, such as Real Time RT-PCR.

To evaluate the suitability of the sandwich hybridization assay on culture samples from *P. pastoris*, a series of cultures were carried out using the *P. pastoris *X-33 derived strain containing the expression vector pPICZFLDαROL (called here X-33 pROL), which expresses the *Rhizopus oryzae *lipase (ROL) under control of the nitrogen regulated P*FLD1 *promoter [5]. This promoter has been used to produce ROL in fed-batch cultures at high cell densities using sorbitol and methylamine as carbon and nitrogen respectively [6], as well as compared in terms of yields and productivities with the classic P*AOX1 *[7].

In order to investigate further whether ROL overexpression triggers the UPR in *P. pastoris*, the following bioprocess-relevant marker genes, involved in protein processing and central metabolism, were selected to monitor their transcriptional levels during fed-batch cultivations: i) the ***KAR2 ***gene [GenBank accession number: AY965684] encoding the BiP protein, a chaperone of the HSP70 class that plays an important role in the unfolded protein stress response (UPR) [8,9]. Heterologous production of human trypsinogen in *P. pastoris *during fed-batch cultivations has been observed to be accompanied by an increase of intracellular levels of BiP [10]; ii) the protein disulphide isomerase gene (***PDI***) [GenBank accession number: AJ302014], which codes for a lumenal ER enzyme that catalyses the mechanism of disulphide bond formation [11]; iii) the ***AOX1 ***gene [GenBank accession number: U96967], coding for the alcohol oxidase enzyme AOX, the first enzyme in the methanol oxidation pathway, which is highly induced in methanol growth conditions but strongly repressed on glycerol or glucose culture conditions [2]; iv) the ***FLD1 ***gene [GenBank accession number: AF066054], which is responsible for the synthesis of the formaldehyde dehydrogenase enzyme [12], an enzyme implied both in the methanol oxidation pathway and methylamine assimilation metabolism; v) the ***26S *rRNA **[GenBank accession number: D43818], which represents a very highly populated molecule with about 200,000 molecules in exponentially growing cells and a close relationship to the specific growth rate [3,13]; and vi) the product-encoding gene (***ROL***) [GenBank accession number: AF229435].

The following series of cultivations were carried out: First, a series of preliminary shake flask experiments was performed. Three different carbon sources were used, glycerol as a repressing carbon source, methanol as an inducing carbon source and sorbitol as a non-repressing carbon source of the P*FLD1 *promoter, respectively. Methylamine was used as inducing nitrogen source in the cultures containing methanol and sorbitol. Dithiothreitol (DTT) was added in a control culture containing glycerol and ammonium sulphate. DTT is a reducing agent that inhibits disulphide bonds formation, thus preventing the correct folding of proteins in the ER and subsequently inducing the Unfolded Protein Response, UPR [8,9].

Second, after these preliminary experiments, a series of fed-batch fermentations were carried out at two different specific growth rates, namely under growth-limiting conditions (0.01 h^-1^) and under carbon-excess conditions (about 0.02 h^-1^).

Third, an additional fed-batch fermentation was performed with the genetically modified *P. pastoris *strain GS115H co-expressing constitutively the *S. cerevisiae HAC1 *gene, coding for a general UPR transcription factor [14], as well as the ROL gene under the control of the *FLD1 *promoter.

## Results and discussion

### Preliminary shake flask experiments

**Figure 1:**
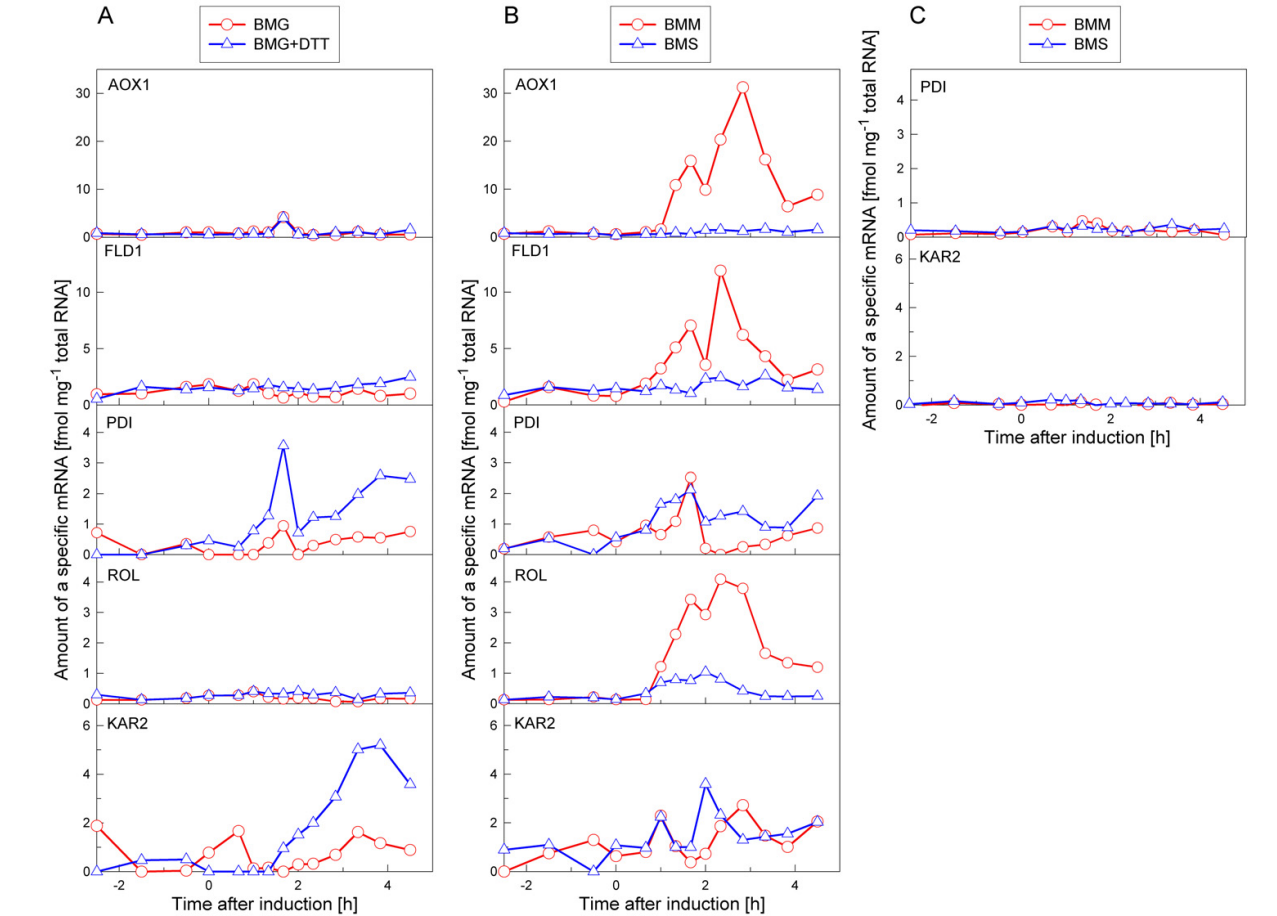
**Transcriptional analysis in shake flask cultures of the *P. pastoris *X-33 pROL**. Transcriptional levels of *AOX1, FLD1, PDI, ROL *and *KAR2 *expressed as fmol of mRNA per μg of total RNA. Time = 0 hours corresponds to change of cells from BMG medium to the induction medium. (A) Cells grown on BMG and BMG+DTT media; (B) Cells grown on BMM and BMS media. (C) Control cultivation: X-33 cells grown on BMM and BMS media.

A battery of shake flask cultures was initially performed in order to test the functionality of the designed probes and the transcriptional response of the genes of interest upon induction of ROL expression. Thus, cells were grown in minimal medium containing different combinations of carbon (glycerol, methanol or sorbitol) and nitrogen sources (ammonium or methylamine). In addition, cells were grown in the presence of DTT, as a positive control for UPR-triggered cells. The results of the transcriptional analysis of the *AOX1*, *FLD1*, *PDI*, *ROL *and *KAR2 *genes are summarised in figure 1.

As expected, in the culture containing methanol as a sole carbon source (BMM), the level of *AOX1 *mRNA increased soon after the addition of methanol into the medium, reaching more than 30 fmol μg^-1 ^of total RNA. Such drastic increase is consistent with the fact that the *AOX1 *promoter is one of the strongest promoters known in yeast (*AOX1 *mRNA levels in methanol-grown *P. pastoris *cells can reach about 5% of the total cell's mRNA, [15]). As also expected, the glycerol-grown cells did not show any significant *AOX1 *induction.

The *FLD1 *mRNA showed a similar profile as the *AOX1 *mRNA, i.e. it increased 40 to 60 min after induction in the BMM culture, reaching about 12 fmol μg^-1 ^of total RNA, whereas in the BMG and BMG+DTT cultures only basal levels of *FLD1 *mRNA were observed. The maximum level of *FLD1 *mRNA in the BMS culture, containing methylamine, was significantly lower than the corresponding value in the BMM culture, confirming the synergistic effect of methanol and methylamine on *FLD1 *induction.

The pattern of the *ROL *mRNA behaved generally similar to the *FLD1 *mRNA, that is, induction of ROL expression was only detected in BMM and BMS media. In particular, in the BMM culture, *ROL *mRNA levels were three- to fourfold higher than in the BMS culture. However, although *FLD1 *and *ROL *genes are under the control of the same promoter, their corresponding mRNAs levels were significantly different.

*PDI *and *KAR2 *mRNAs showed a clear induction in cells growing on BMG+DTT, indicating that the experimental set-up was appropriate for detection of UPR-related stress at the mRNA level. In addition, induction of *PDI *and *KAR2 *was detected in cells grown in BMM and BMS media, i.e. after induction of ROL. Thus, the parallel up-regulation of the *PDI *and *KAR2 *mRNAs caused by either DTT or ROL further supported the hypothesis that ROL overexpression triggers the UPR in *P. pastoris*.

### Fed-batch cultivations

The specific growth rate has proven to be an important parameter for the productivity of secreted heterologous proteins in the *P. pastoris *system [16,17] and, in particular, when using the P*FLD1 *promoter for heterologous extracellular ROL production [6]. Hence, two fed-batch cultures were performed with the *P. pastoris *X-33pROL expressing ROL under the transcriptional control of the P*FLD1 *promoter according to a previously developed strategy [6], where sorbitol is used as non-repressing carbon source and methylamine is the inducing nitrogen source substrate during the production phase. The induction phase of the first cultivation was performed under growth rate-limiting conditions (μ was kept at about 0.01 h^-1^), whereas a near-μ_max _growth rate of about 0.02 h^-1 ^was maintained under carbon excess conditions during the induction phase of the second cultivation.

#### Fed-batch cultivation of X-33pROL under growth limiting conditions (controlled low μ)

**Figure 2:**
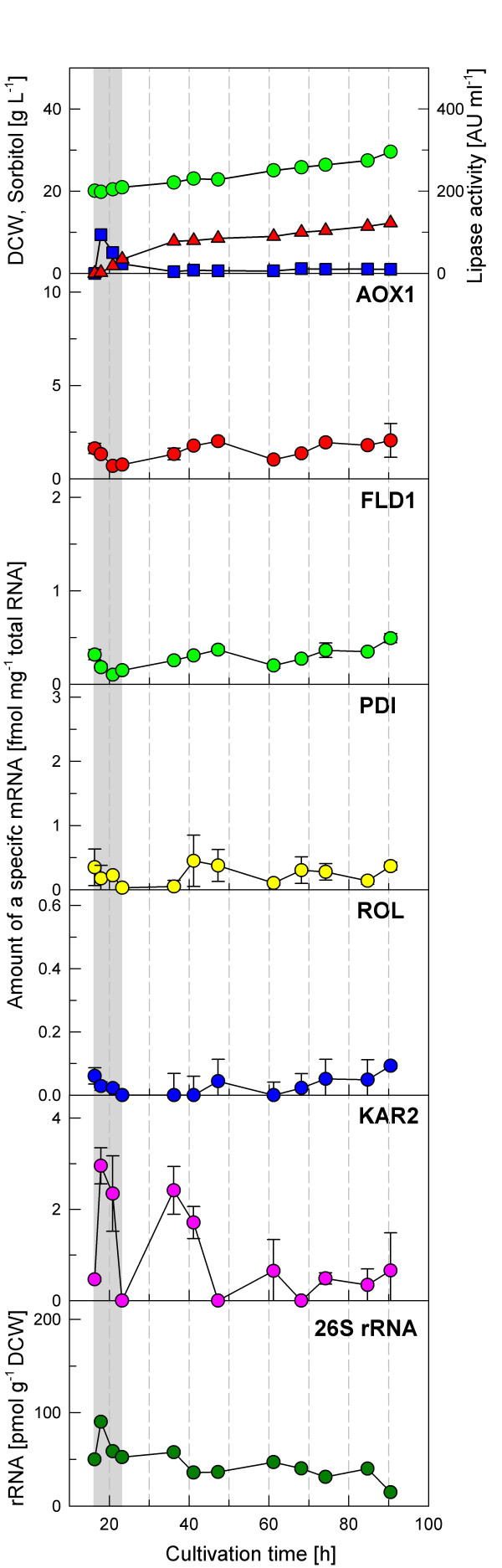
**Transcriptional analysis during a fed-batch cultivation of *P. pastoris *X-33 pROL performed at a controlled specific growth rate of 0.01 h^-1^**. Dry cell weight (green circle), sorbitol concentration (blue square) and extracellular lipase activity (red triangle) are indicated. mRNAs levels are expressed as fmol of each mRNA per μg of total RNA in the sample. 26S rRNA is presented as pmol g^-1 ^DCW.

The corresponding analyses of the mRNAs levels of the five specific genes are depicted in figure 2. The levels of the *AOX1*, *FLD1*, *PDI*, and *ROL *mRNAs showed small variations when the cells were transferred into a medium containing sorbitol and methylamine at growth limiting conditions. Only for *KAR2*, a clear transient induction (about 6-fold) was detected at the early stage of the fed-batch transition phase. This up-regulation lasted for about 20 h. Thereafter the level of KAR2 mRNA decreased steadily towards the basal level which was reached 35 h after induction.

As observed in the preliminary shake flask cultivations, significant transcription levels of the *AOX1 *gene are detected in cells growing on sorbitol + methylamine. This could be due to partial derepression of the *AOX1 *promoter, as sorbitol has been described as a non-repressing carbon source of this promoter [18,19]. However, it could also be due to its partial induction caused by possible formation of methanol from the formaldehyde generated by methylamine metabolism [20], or a combination of both mechanisms.

#### Fed-batch cultivation of X-33pROL under carbon-excess (near μ_max_)

**Figure 3:**
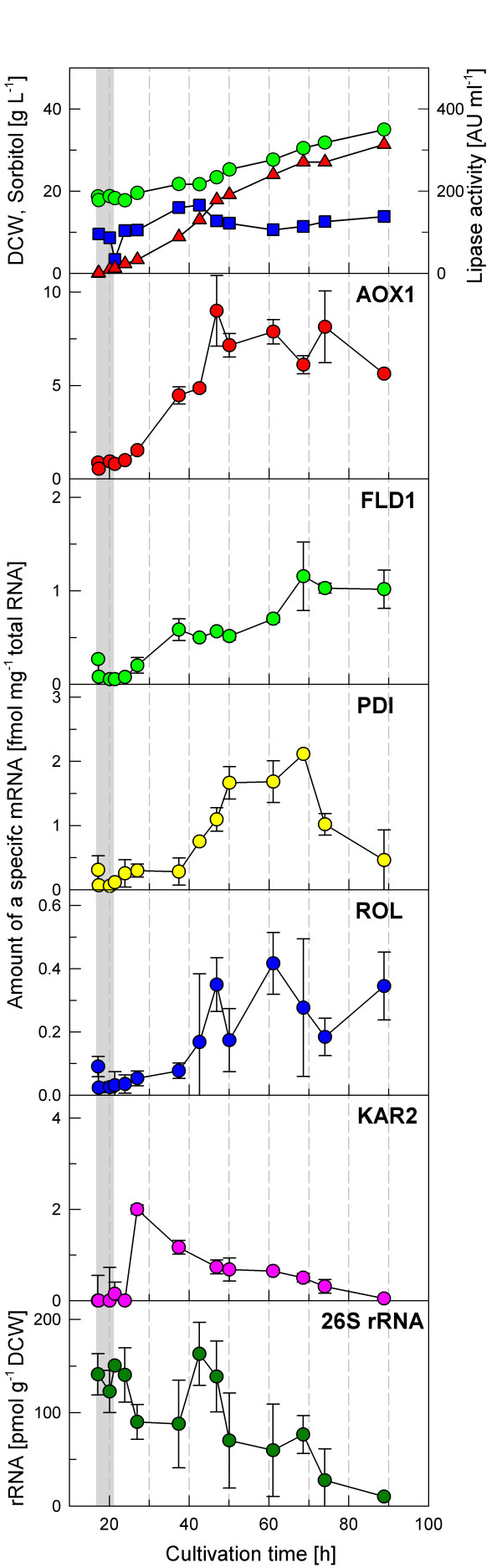
**Transcriptional analysis during a fed-batch cultivation of *P. pastoris *X-33 pROL under carbon excess conditions (mean specific growth rate of about 0.02 h^-1^)**. Biomass (dry cell weight) (green circle), sorbitol concentration (blue square) and extracellular lipase activity (red triangle) are indicated. mRNA levels are expressed as fmol of each mRNA per μg of total RNA in the sample. **26S rRNA is presented as pmol g^-1 ^DCW**.

In the fed-batch cultivation performed under substrate excess conditions, i.e. growing at a near μ_max _of about 0.02 h^-1 ^(figure 3), the mRNA levels of the target genes were clearly different compared to the previous cultivation.

The *AOX1 *mRNA level started to increase steadily soon after the transition phase until 50 hours of cultivation. Thereafter, the level of *AOX1 *mRNA stayed at about 8 fmol μg^-1 ^total RNA, which is a relatively high value compared to the level of the other analysed genes. This further confirmed that the *AOX1 *gene is derepressed and/or partially induced to a significant extent in *P. pastoris *cells growing on sorbitol plus methylamine. Nevertheless, in contrast to the well documented partial catabolite repression of methanol metabolism by different multicarbon compounds observed in other methylotrophic yeast species (e.g. *Hansenula polymorpha*), the level of *AOX1 *mRNA in *P. pastoris *was still markedly lower with sorbitol as a carbon source compared to growth on methanol, as observed in the shake flask experiment where the *AOX1 *mRNA increased to about 30 fmol μg^-1 ^total RNA (figure 1).

The amount of *FLD1 *mRNA increasing continuously after induction, reaching about 1 fmol μg^-1 ^total RNA at 70 h of cultivation. After this point, the level of *FDL1 *mRNA did not change until the end of the cultivation. *PDI *and *ROL *mRNAs showed similar profiles. Both genes were increasingly induced until 60 h (*ROL*) and 70 h (*PDI*) of cultivation. However, while the *PDI *mRNA level clearly decreased soon after this point down to almost the basal level towards the end of the cultivation, the decrease in the last part of the cultivation was not so pronounced for the ROL mRNA. The profile of *KAR2 *mRNA was completely different from the other analysed mRNAs. Similar to the growth-limited fed-batch cultivation, there is a sharp increase in the *KAR2 *mRNA level shortly after induction followed by a constant decrease, reaching basal levels towards the end of the cultivation.

#### Fed-batch cultivation of GS115HpROL under carbon-excess conditions (near μ_max_)

**Figure 4:**
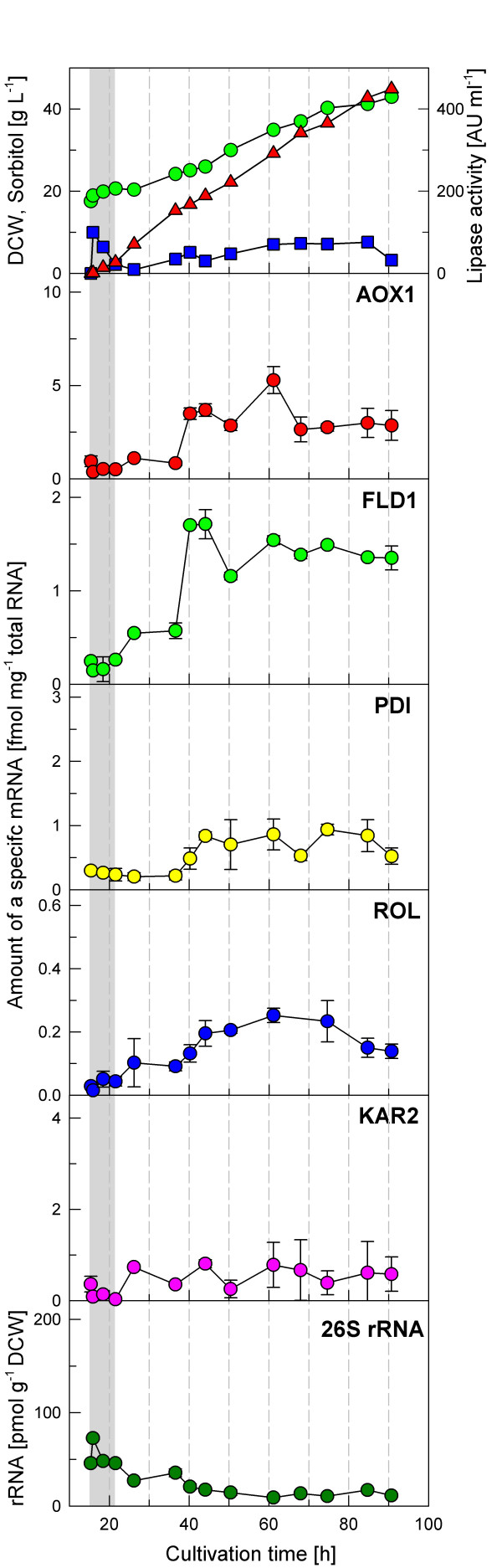
**Transcriptional analysis during a fed-batch cultivation of *P. pastoris *GS115H/HAC1 pROL under carbon excess conditions (mean specific growth rate of about 0.02 h^-1^)**. Biomass (dry cell weight) (green circle), sorbitol concentration (blue square) and extracellular lipase activity (red triangle) are indicated. Analysed mRNAs are expressed as fmol of each mRNA per μg of total RNA in the sample. 26S rRNA is presented as pmol g^-1 ^DCW.

A third fed-batch fermentation was carried out with the *P. pastoris *strain GS115H pROL, containing both the pGAPHAC1 and pPICZFLDαROL plasmids, under carbon-excess conditions, i.e. at a near-maximum specific growth rate of about 0.02 h^-1^. The cultivation data are shown in figure 4. Also here the level of the *AOX1 *mRNA slowly increased when cells were switched from glycerol + ammonium to sorbitol + methylamine, indicating a similar derepression (and/or partial induction) pattern of the *AOX1 *gene as in the fed-batch culture of X-33 pROL. However, in GS115H pROL the maximum of *AOX1 *mRNA was significantly lower (5.3 ± 0.71 fmol μg^-1 ^total RNA), compared to the maximum reached in the cultivation of X-33 pROL (8.9 ± 1.82 fmol μg^-1 ^total RNA).

In contrast, the mean *FLD1 *mRNA level along the induction phase was higher than in the corresponding cultivation with X-33 pROL. However, the mean level of the *ROL *mRNA was slightly lower than in the X-33 fed-batch culture, again suggesting significant differences in the mRNA stability of the *FLD1 *and *ROL *transcripts. Notably, the maximum specific lipase activity in the GS115H pROL culture was higher than in the culture of X-33 pROL, 10928 AUper g cell dry weight versus 8008 AU per g cell dry weight, respectively. Considering that extracellular product proteolytic activity was undetectable (both by western blot analysis and protease activity assays under the tested growth conditions) [6], this observation may be explained by an improved efficiency of the protein folding and secretion machinery in the GS115H strain. This improved efficiency could be the result of the combination of lower ROL synthesis rates with pre-conditioning of the cells to ER stress by the higher level of HAC1 in this strain.

On the other hand, an unexpected behaviour was observed in the mRNA levels of *PDI *and *KAR2*. In principle, the constitutive overexpression of the *HAC1 *gene leads to upregulation of the synthesis of more than 300 genes involved in the UPR, including *KAR2 *and *PDI *[21]. However, the basal *KAR2 *or *PDI *mRNA levels measured at the end of the batch phase of the cultivation of the HAC1-overexpressing strain GS115H pROL, were essentially the same as those measured for the X-33 strain at the same stage of the cultivation. Moreover, the *PDI *mRNA level stayed lower during the whole cultivation compared to the cultivation of X-33. The maximum in GS115H pROL was reached after 44 h. Thereafter the PDI mRNA level stayed at about 0.9 fmol μg^-1 ^total RNA until the end of the cultivation. The *KAR2 *mRNA showed had a similar pattern, i.e. its level was relatively constant along the whole induction phase, below 1 fmol μg^-1 ^total RNA. This was significantly lower than the *KAR2 *mRNA level observed in the fermentations with X-33 pROL.

Overall, these results suggest that the constitutive expression of the *S. cerevisiae *HAC1 gene in *P. pastoris *allows for a "pre-conditioning" of the host cells, which results in a lower expression of UPR-related genes (*PDI *and *KAR2*) upon ROL induction. Nevertheless, further systematic studies are needed at the transcriptional level to unravel the complex physiological dynamic responses of *P. pastoris*.

#### *26S *rRNA analyses

Figures 2 to 4 show the 26S rRNA profiles in the performed *P. pastoris *fed-batch cultures. The 26S rRNA levels normalized by the cell dry weight of the samples show a significant decrease over the cultivation time. Considered that the major fraction of total RNA is ribosomal RNA, 26S rRNA represents the ribosome amount which is correlated with the specific growth rate [3,13]. Comparison of the two cultivations of the X-33 pROL strain indicates a clearly higher 26S rRNA level if the strain is grown at a higher feed rate, however the 26S rRNA level clearly decreased at the time when ROL was strongly expressed. Although the same growth rate was kept in the cultivation with the GS115H pROL strain (overexpressing HAC1), the 26S rRNA level was significantly lower in this case, suggesting that HAC1 constitutive overexpression may affect the cellular maintenance and result in a downregulation of ribosomal RNA synthesis and, therefore in protein synthesis, which however needs further investigation.

## Conclusion

In this study the influence of the specific growth rate, carbon and nitrogen sources and the induction of ROL expression on the transcriptional level of a reduced set of bioprocess-relevant genes has been quantitatively analysed. The bead-based sandwich hybridization assay has proved to be a reliable instrument for quantification of specific mRNA species in *P. pastoris *fermentation samples, although further improvement of cell lysis protocols and probe design for some specific genes is needed.

Clearly, the specific growth rate was a key factor in the evolution of the transcriptional levels of the analysed genes following induction of ROL expression. The mRNA levels of the analysed set of genes were generally higher at higher growth rates. ROL secretion levels are also higher at higher growth rates. These results strongly suggests that transcription appears to be the major limiting factor in extracellular ROL production under growth-limiting conditions (0.005 to 0.01 h^-1^).

Besides, we observe that ROL overexpression in the X-33-derived strain clearly induces the expression of UPR-related genes such as *KAR2 *and *PDI*. Notably, the induction levels of these marker genes are significantly lower when ROL is overexpressed in a strain having the UPR constitutively activated (GS115H). Additionally, although *ROL *mRNA levels in this strain are not higher than in the corresponding cultivation with the X-33 derived strain, higher extracellular product titers are achieved, suggesting that under carbon excess conditions (i.e. near μ_max_) secretion or folding efficiency, but not transcription, is a major bottleneck in the extracellular production of ROL in the X-33 derived strain.

Thus, our results suggest that the improvement in ROL production observed with the GS115H strain is not provoked by an upregulation of ROL transcription, but by an improvement in secretion or folding efficiency. Also, protein folding and secretion appear to be a limiting step for extracellular ROL production in cells growing at high specific growth rates. This interrelation between specific growth rate and protein synthesis and secretion rates is consistent with recent studies in fungi [22].

Besides, this study points at a complex response to both dynamic environmental conditions intrinsic to fed-batch cultivation and endogenous stress factors (ROL overexpression). The interaction/interconnection between environmental and endogenous factors in relation to UPR needs further systematic studies in order to understand its dynamics (e.g. *KAR2 *and *PDI *transcription profiles).

From the bioprocess point of view, the *P. pastoris *strain expressing the *S. cerevisiae HAC1 *geneconstitutively seems to be a promising cell engineering strategy applicable to ROL production although further studies are needed to fully evaluate its potential. Notably, the same engineering strategy has proven to improve moderately (1.3 fold) the secretion efficiency of an antibody fragment, which also triggers the UPR [12].

## Methods

### Strains

A *P. pastoris *X-33-derived strain containing the expression vector pPICZFLDα ROL [5] integrated in its genome's *FLD1 *locus was used throughout this study (called X-33 pROL). Besides, we also used a *P. pastoris *GS115H strain constitutively overexpressing the *S. cerevisiae HAC1 *gene [13] co-transformed with the vector pPICZFLDα ROL (called GS115H pROL). *P. pastoris *X-33 and GS115 are isogenic strains except for GS115's histidine auxotrophy; in particular, the X-33 strain is a His^+ ^derivative of strain GS115 generated by transformation of the latter strain with a DNA fragment containing the *P. pastoris his4 *gene [23].

### Culture maintenance

*P. pastoris *strains were grown on YPD agar medium plates containing (w/v): 1% yeast extract, 2% peptone, 2% dextrose, 2% agar and stored at 4°C. Long-term stocks were prepared as recommended by Invitrogen Corporation (Carlsbad, CA, US) and deep frozen at -80°C.

### Inoculum preparation

Pre-inoculums for shake-flask and bioreactor cultures were grown for 24 h in baffled shake flasks at 30°C, 250 rpm, in BMGY (Buffered Glycerol-Complex Medium) containing 1% (w/v) yeast extract, 2% (w/v) peptone, 100 mM potassium phosphate, pH 6.0, 40 mg L^-1 ^biotin, 1% (w/v) glycerol in 50 mL of final volume. Cells were centrifuged at 4000 × g and resuspended in the required fresh medium to inoculate shake flask cultivations. Alternatively, the 50 mL culture was used to inoculate 500 mL (final working volume) of BMGY in a 1-liter bench-top bioreactor (Braun Biotech, Melsungen, Germany). The culture was grown overnight at 30°C and subsequently was centrifuged at 4000 × g. Harvested cells were resuspended in bioreactor culture medium and used to inoculate a 5-liter Biostat ED bioreactor (Braun Biotech, Melsungen, Germany).

### Shake flask cultures

Four shake flask experiments were performed in parallel in 500 mL shake flasks containing 50 mL of medium. Shake flasks were inoculated from a pre-culture of the *P. pastoris *X-33 strain containing the pPICZFLDα ROL expression vector grown in BMG (buffered minimal glycerol) medium. Four different media were used: (i) BMG medium containing 1% (w/v) glycerol, 1.34% YNB without aminoacids, 40 mg L^-1 ^biotin and 100 mM potassium phosphate pH 6.0; (ii) BMG + DTT includes the same components as BMG plus addition of 10 mM DTT; (iii) BMM (buffered minimal methanol) containing 0.5% (w/v) methanol, 1.34% YNB without aminoacids and ammonium sulphate, 0.4% (w/v) methylamine hydrochloride, 40 mg L^-1 ^biotin and 100 mM potassium phosphate pH 6.0; (iv) BMS (buffered minimal sorbitol) contained 1% (w/v) sorbitol, 1.34% YNB without aminoacids and ammonium sulphate, 0.4% (w/v) methylamine hydrochloride, 40 mg L^-1 ^biotin and 100 mM potassium phosphate pH 6.0. All incubations were performed at 30°C and 150 rpm. The cultures were grown for 8 hours, and 1 mL samples were withdrawn every 20 min. The collected samples were immediately added into a tube containing 100 μL of cool inhibition solution (ethanol: phenol 95:5 v/v), centrifuged for 1 min at 12,000 rpm with a bench top centrifuge, the supernatant was discarded and the pellet was resuspended in 1 mL of RNALater (Ambion) and stored at -70°C.

### Fed-batch cultivation set up and operational conditions

Fed-batch cultivations were performed using a mineral medium with the following basal composition (per litre): KH_2_PO_4 _12.0 g, MgSO_4_·7H_2_O 4.70 g, CaCl_2_·2H_2_O 0.36 g, antifoam (alcoxylated ester JG73, Strucktol, Hamburg, Germany) 0.1 mL, 1 mL of a biotin solution (400 mg L^-1^), and 1 mL of a trace salts solution (0.2 mM CuSO_4_·5H_2_O, 1.25 mM KI, 4.5 mM MnSO_4_·4H_2_O, 2 mM Na_2_MoO_4_·2H_2_O, 0.75 mM H_3_BO_3_, 17.5 mM ZnSO_4_·7H_2_O, 44.5 mM FeCl_3_·6H_2_O). The biotin and trace salt solutions were sterilized separately by microfiltration (Millex GS 0.22 μm, Millipore, Malsheim, France).

Fed-batch cultures were carried out in a 5-litre Braun Biostat ED bioreactor (B. Braun Biotech, Melsungen, Germany), with an initial working volume of 3.5 L. The cultivation process comprised three phases: Firstly, a batch phase with glycerol (40 gL^-1^) as carbon source and ammonium sulfate at the corresponding stoichometric quantity (9.2 g L^-1^) as sole nitrogen source. Secondly, after glycerol depletion, a batch of 10 g L^-1 ^of sorbitol and 3 g L^-1 ^of methylamine (CH_3_NH_2_·HCl) was added into the bioreactor to induce the P*FLD *promoter. This transition phase lead to the third phase of the cultivation, i.e. the induction phase, which consisted in the exponential feed of sorbitol and methylamine as sole carbon and nitrogen sources, respectively. The feeding stock solution contained 300 g L^-1 ^of sorbitol and 35 g L^-1 ^of methylamine, and was added to the reactor by an automatic microburette MicroBU-2031 from Crison Instruments (Alella, Barcelona, Spain). The cultivation conditions were: stirring rate 800 rpm, temperature 30°C, pH controlled at 5.5 by addition of 5 M KOH. Dissolved oxygen was controlled above 30% with an air flow rate between 1.5 and 20 L min^-1^.

A pre-programmed exponential feeding rate strategy was designed with the objective to control the specific growth rate at a constant value along the fed-batch induction phase [6]. For RNA analysis, 200 to 500 μL samples were withdrawn, depending on the cell density and the same treatment as for the shake flask samples was carried out.

### Analysis of glycerol, sorbitol, methylamine and ammonium

Glycerol and sorbitol concentrations were analysed by HPLC with an HP 1050 liquid chromatograph (Hewlett Packard, Palo Alto, CA, USA) using an Aminex HPX-87H ion-exchange column from Bio-Rad. The mobile phase was 15 mM sulphuric acid and the injection volume was 20 μL. Data were analysed by the Millenium 2.15.10 software (Waters Corporation, Mildford, MA, USA).

Methylamine was analysed by HPLC (Hewlett Packard 1090) with a UV-Vis diode array detector using a Hypersil AA-ODS column (Hewlett Packard, Palo Alto, CA, USA) with a Hypersil ODS Guard pre-column. Solvent A: 20 mM sodium acetate, 0.3% (v/v) tetrahydrofurane (THF), 0.018% (v/v) triethilamine (TEA); Solvent B: 100 mM sodium acetate, 40% (v/v) methanol, 40% (v/v) acetonitrile.

The ammonium concentration was analysed using a colorimetric method (LCK302 kit, Dr. Lange, Düsseldorf, Germany) with an RSD of about 8% in the concentration range used.

### Lipase activity

Lipase activity was determined using the Lipase colorimetric assay (LIP kit, reference no. 1821792, Roche Diagnostics, Mannheim, Germany) [5].

### Sample preparation

Samples from shake flask cultures and fed-batch fermentations were withdrawn and immediately added in a tube containing 100 μL of ice-cool inhibition solution (ethanol:phenol 95:5 v/v) and centrifuged for 1 min at 12,000 rpm using a refrigerated benchtop centrifuge at 4°C. Supernatants were carefully removed by a water vacuum pump and the cell pellets were resuspended in 1 mL of RNALater (Ambion) and stored at -70°C.

### Yeasts lysis and RNA extraction

Total RNA extractions from yeast samples were performed using the RNA extraction kit (Qiagen) according to the manufacturer instructions. Samples were diluted in DEPC water to achieve the same optical density and cells were disrupted with a FastPrep cell homogenizer (ThermoSavant). Total extracted RNA was quantified using the RiboGreen RNA Quantification Kit (Molecular Probes). Ribosomal RNA (16S and 23S rRNA from *E. coli; *component C) was used as standard for total RNA quantification assays.

### Oligonucleotide probes design and synthesis

Primers for DNA amplification and oligonucletide probes for sandwich hybridization were designed using the software Clone Manager 5.0 (SE Central). Oligonucleotides were synthesised by Sigma Genosys. Capture probes were 5' biotin labelled by Sigma Genosys and detection probes were labelled with digoxigenin at 3' using the DIG Oligonucleotide Tailing kit (Roche) according to the manufacturer's instructions. The primers and probes designed and used in this study are summarized in table 1.

**Table 1:** Primers and probes used in this work.

	*Sequence 5' → 3'*	*Position*	*Tm °C*	*Th °C*
***AOX1***				
Forward primer	AGC AGG TGA GAA CAA CCT CAA C	583	66	
Reverse primer	AAG GTC CAC CGT AGG CAT TAG A	1988	66	
Detection	ACC ATT GGC GTA CCA TTG	1510	54	55
Capture	GAA TCT CAG CAT CAC CAC	1475	54	55
Helper	GAC TCG TAC TGA GGC TTG	1440	56	55
***26S rRNA***				
Forward primer	AAT GAG TGG TGA CAC GAG AC	8	60	
Reverse primer	GGA TGT CGG AGA TGC GTA G	168	62	
Detection	GCG TAG ACG CAC CAA CT	155	54	50
Capture	ACT CCT CCT ACG CCT TC	61	54	50
Helper	TTG GCC GTC TCG TGT CA	33	54	50
***PDI***				
Forward primer	TGT CCG CTC TCA CAC TAG CA	726	62	
Reverse primer	TTC CTC GAC TGG TCT GAG TG	2182	62	
Detection	CCG ACT AGC TTG AAG ACT	1827	54	55
Capture	CCT CTC CTG CGA TGA ATT	1760	54	55
Helper	TCT CAA TCA GCT CGG TCA	1742	54	55
***FLD1***				
Forward primer	TCA CTG GTG TAT GCC ACA CT	839	60	
Reverse primer	GAT GTT GTC CAG GTC ATG TC	1788	60	
Detection	AGC AGT GTT GAT AGC AGC AC	1278	60	55
Capture	T AG GAG CTT CGT CTT GGA CT	1213	60	55
Helper	TAA CCA CT GAGA TGT CAG CC	1192	60	55
***KAR2***				
Forward primer	GCC ATG CTA TTG GTC GTA GT	49	60	
Reverse primer	GGT AGG CTC GTT CAC AAT TC	633	60	
Detection	TTG AAG TAG GCT GGA ACG GT	554	60	50
Capture	GTG GCT TGA CGT TGA GCG T	575	60	50
Helper	CGA TGA GAC CGG CAT CCT T	595	60	50
***ROL***				
Forward primer	CTG ATG GTG GTA AGG TTG T	1271	56	
Reverse primer	AAG GAA CGA TAG AGT TAC TG	2020	56	
Detection	TGC ACC ACC GAG TGA GTG A	1716	60	50
Capture	AGA TCC ATA CCG GCA AGC AA	1742	60	50
Helper	ACA GTC TTG GTT CAC GTT GG	1765	60	50

Tm: melting temperature. Th: hybridisation temperature. Position: First nucleotide localization within the corresponding gene sequence at GeneBank

### Synthesis of *in vitro *transcripts for standard values

For *in vitro *transcription, the specific PCR products (see Table 1) containing the T7 promoter sequence were used as template for *in vitro *transcription performed according to the MAXIscript protocol (Ambion). The concentrations of the *in vitro *transcripts were determined by measuring the absorbance at 260 nm and using Ribogreen RNA quantification assay (Molecular Probes). To calculate the molecular weight of each transcript the web-based Biopolymer Calculator of the Schepartz Laboratory of Chemical Biology (Yale University, US) was used. The *in vitro *transcripts were diluted in DEPC water and used as standards in sandwich hybridization assays.

### Sandwich hybridization assay

In the sandwich hybridization assay, the target RNA is hybridized with three DNA probes (see Table 1), complementary to the target RNA sequence. A capture probe is labelled with biotin at its 5' site, which immobilizes the target RNA on a streptavidin-coated MagneSphere particle (Promega). Prior to sandwich hybridization, the detection probe is tailed with digoxigenin. Anti-DIG-alkaline phosphatase Fab-fragments (Roche) are added to the reaction after hybridization and the enzymatic reaction is performed with BBTP (2'-[2-benzothiazoyl]-6'-hydroxybenzothiazole phosphate substrate, AttoPhos, Promega), which is cleaved to inorganic phosphate (Pi) and the fluorescent product BBT (2'-[2-benzothiazoyl]-6'-hydroxybenzothiazole). The signal is detected by a fluorescence reader (Victor2, Wallac Instruments) following to the manufacturer's instructions.

The sandwich hybridization was carried out in 96-well plates, using a Thermomixer Comfort incubator (Eppendorf). The total volume of the hybridization reaction was 100 μL, containing 5 × SSC buffer (0.15 M sodium chloride, 0.015 M sodium citrate, pH 7.0), 20% (v/v) formamide, 2% (v/v) Denhardts reagent, 3% (v/v) dextran sulphate and 0.2% (v/v) SDS. An amount of 5 pmol of biotin-labelled capture probe, 5 pmol of helper probe and 1 pmol of dig-tailed detection probe were added to the hybridization solution. Finally, 10 μL of the specific mRNA *in vitro *standards or extracted RNA from samples were added to each well.

The hybridization took place at 50 or 55°C, for 30 min at 700 rpm shaking. After hybridization, 20 μL of streptavidin-coated magnetic beads (Promega) were added to the wells, followed by incubation at 37°C for 30 min and 500 rpm. Before addition, the magnetic particles were washed three times with SSC buffer and finally taken into the original volume with same buffer. For immobilisation of the target RNA, the MagnaBot 96 Magnetic Separation device (Promega) was used to separate the beads from the solution. The magnetic beads were washed twice with 130 μL of SSC buffer containing 0.05% (v/v) SDS by incubating the plates at 25°C for 2 min and 700 rpm shaking. After washing the beads, 100 μL of Anti-DIG-alkaline phosphatase FAB fragments diluted 1:2000 in SSC buffer were added to each well and incubated at 25°C for 30 min and 700 rpm shaking. To remove the unbound antibody, the wells were washed four times with SSC buffer containing 0.05% (v/v) SDS by incubating at 25°C for 2 min and 700 rpm shaking. After the washing steps, the beads were transferred to a new microplate and washed again. After removing the washing buffers, 100 μL of Attophos fluorescent substrate for alkaline phosphatase was added to the wells and incubated at 37°C and 700 rpm shaking for 20 min.

The reaction was stopped by separating the beads from the solution using the MagnaBot 96 Magnetic Separation device. The supernatant containing fluorescent compound was transferred to a black microplate and measured using the Victor 2 fluorescence reader at an excitation wavelength of 430 nm and an emission wavelength of 560 nm.

Fed-batch samples were analysed in duplicates, while for shake flask experiments only one run per sample was performed due to the limited sample volume. The standard deviation of the assay for each one of the probes was as follows: *AOX1*: ± 0.32, *FLD1*: ± 0.062; *PDI*: ± 0.15; *KAR2*: ± 0.301; *26SRNA*: ± 1432.79. For each series of samples, a calibration curve using the *in vitro *synthesised standards for each specific mRNA species was performed and the amount of a given mRNA species was calculated based on the linear part of the resulting standard curve.

The amounts of the given mRNA species obtained from the sandwich hybridization assay are expressed in fmol of each specific mRNA, and are normalized by the total RNA of the corresponding sample (fmol μg^-1^). The amount of 26S rRNA is presented as pmol g^-1 ^DCW.

## Authors' contributions

D.R. performed the fermentations and the sandwich hybridization assays, as well as manuscript preparation. P.N. and M.B. contributed in the design and supervision of the mRNA analyses. N.K.K. contributed to some of the sandwich hybridization assays. F.V. and P.F. contributed in the overall design of this study and DR's project supervision. P.N., M.B., F.V. and P.F. also collaborated in the results discussion and manuscript preparation.
